# Self-Assembled Tetrahedral [Cr^III^_4_L_6_]^12+^ Cage Displaying Near-Infrared Spin-Flip Photoluminescence

**DOI:** 10.1021/acs.inorgchem.4c04180

**Published:** 2024-11-28

**Authors:** Yating Ye, Carlos M. Cruz, Benjamin Doistau, Enrique Colacio, Claude Piguet, Juan Manuel Herrera, Juan-Ramón Jiménez

**Affiliations:** † Department of Inorganic Chemistry, 16741 University of Granada and “Unidad de Excelencia en Química (UEQ)”, Avda. Fuente Nueva s/n, Granada 18071, Spain; ‡ Department of Organic Chemistry, 16741 University of Granada and “Unidad de Excelencia en Química (UEQ)”, Avda. Fuente Nueva s/n, Granada 18071, Spain; § Laboratoire de Chimie et de Biochimie Pharmacologiques et Toxicologiques (UMR 8601), Université Paris Cité, CNRS, 45 rue des Saint-Pères, Paris F-75006, France; ∥ Department of Inorganic and Analytical Chemistry, 27212 University of Geneva, 30 quai E. Ansermet, Geneva 4 CH-1211, Switzerland

## Abstract

The thermodynamically controlled self-assembly of bis-bidentate quaterpyridine ligand, L = 2,2′:5′,5″:2″,2‴-quaterpyridine, with Cr^II^ and subsequent oxidation to Cr^III^ yields the first photoluminescent tetrahedral [Cr^III^_4_L_6_]^12+^ molecular cage. Single-crystal X-ray diffraction reveals the presence of two homochiral cages (ΛΛΛΛ and ΔΔΔΔ) in the unit cell that crystallize as a racemic mixture. Additionally, a PF_6_ anion is observed inside the cavity, in line with isostructural cages built with Ni^II^ or Fe^II^. Each corner of the polyhedron is occupied by weakly antiferromagnetically coupled {Cr­(bipy)_3_}^3+^ (bipy = 2,2′-bipyridine) patterns, as revealed by magnetometry. Upon light excitation in the UV–vis region, spin-flip luminescence from the ^2^E/^2^T_1_ excited states with a maximum at 727 nm (13755 cm^–1^) was detected at room temperature. The measured excited state lifetime of 183 μs is longer than the 102 μs recorded for the mononuclear [Cr­(bipy)_3_]^3+^ complex under anaerobic conditions, whereas the luminescence quantum yields are in the same order of magnitude and amount to 10^–2^ %. The photoluminescence brightness, *B*, calculated using the maxima of the absorption spectra for both species, goes from 14 M^–1^·cm^–1^ for the mononuclear compound to 90 M^–1^·cm^–1^ for the tetrahedral cage. This 6-fold improvement is observed across the entire excitation wavelength range, and it is due to the incorporation of four light-harvester units in the molecular cage.

## Graphical abstract

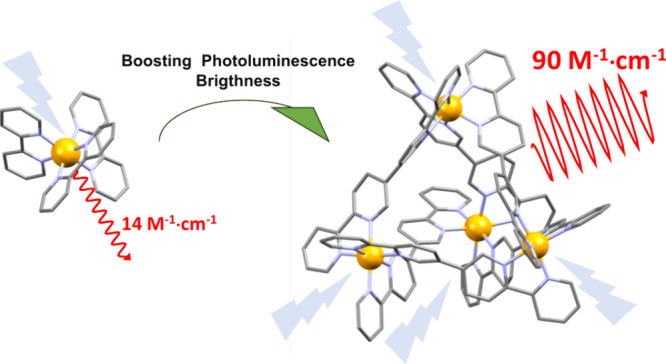

## Introduction

Chromium­(III) polypyridyl complexes have garnered special attention in the past years in the field of photophysics and photochemistry.^1–8^ Cr^III^ ions present an efficient Ruby-like spin-flip emission,^9^ arising from the ^2^E/^2^T_1_ doublet excited state to the ^4^A_2_ quartet ground state,^10–17^ when they are embedded in a strong ligand field environment with a slightly distorted octahedral geometry.^18^ This radiative transition typically occurs in the 700–800 nm range of the electromagnetic spectrum. However, recent advancements in the fine-tuning of the nephelauxetic effect in Cr^III^ complexes have shifted this emission into the NIR-II region, opening new avenues for applications.^19–21^ A notable characteristic of the spin-flip transition is its intraconfigurational nature, involving electronic reorganization within the t_2g_ orbitals, which results in very sharp emissions akin to the f-f transitions in lanthanides. Additionally, these emissions are long-lived, reaching the millisecond range, due to their spin and Laporte forbidden character.^22,23^ These unique properties have led to these complexes to be used for molecular upconversion,^24–28^ molecular thermometry,^17^ circularly polarized luminescence (CPL)^27,29–32^ and photocatalysis.^5,33^ Despite these intriguing properties, there are limited examples of Cr^III^-based metallosupramolecular species, likely due to the complex synthetic procedures required by the inert nature of Cr^III^ ions (ligand self-exchange kinetic constant: *k*_H_2_O_ = 2.4 × 10^–6^ s^–1^ for [Cr­(H_2_O)_6_]^3+^).^34^ Nonetheless, remarkable examples have been reported by Winnpenny and co-workers in which they employed high-temperature solvothermal synthesis to overcome kinetic barriers leading to the serendipitous formation of supramolecular rings and metallacrown wheels.^35–37^ Alternatively, a more rational synthetic approach has been recently developed involving the use of inert Cr^III^ complexes as molecular building blocks to construct photoactive d–d and d–f dinuclear and trinuclear species.^38–40^ Back at the turn of the century, Piguet and co-workers employed the labile Cr^II^ ion to thermodynamically control the self-assembly of metallosupramolecular architectures. Subsequent oxidation of the Cr^II^ to Cr^III^ “locks” the assembly, allowing the obtention of inert Cr^III^-based helicates.^24,41–43^ Analogous to Cr^III^ complexes, low-spin Co^III^ also forms kinetically inert complexes due to the maximized crystal-field splitting stabilization, and they have been employed for the preparation of tetrahedral capsules and helicates using an “assembly-followed-by-oxidation” protocol from a Co^II^ precursor.^44,45^ This approach that possesses advantages of reversible and self-healing self-assembly reactions gives structures that possess a covalent-like level of kinetic robustness. This is usually achieved by using nonlabile metal ions, most commonly the scarce and expensive 4d- and 5d-block elements,^46^ which become dynamic only at high temperatures. Within this context, Lusby and co-workers encapsulates the γ-emitting [^99m^Tc]­TcO_4_^–^ anion within the kinetically inert Co^III^_4_L_6_ tetrahedral cage.^47^ *In vivo* imaging of the molecular composite shows a significant change in biodistribution compared to the free oxo-anion, suggesting the potential applications of supramolecule species in clinical treatments. Other discrete supramolecular cationic tetrahedral cages or capsules with the general formula M_4_L_6_, where L represents 2,2′:5′,5″:2″,2‴-quaterpyridine and derivatives and M denotes various metal ions such as Ni^II^, Co^III^, and Fe^II^, have been extensively studied.^47–52^ Metal ions occupy the vertices of the polyhedral cage, creating central cavities that enable host–guest chemistry and selective anion binding within these systems. For instance, Brechin and co-workers demonstrated some fine-tuning of the magnetic exchange interactions between a Ni^II^_4_L_6_ cage and encapsulated paramagnetic tetrahalometallates.^51^ Other examples based on the labile Fe^II^ was shown to encapsulate selectively PF_6_^–^ over BF_4_^–^.^49^ However, the use of luminescent and inert Cr^III^ ions to form metallosupramolecular capsules or cages remains, to the best of our knowledge, unexplored. The versatile coordination chemistry of Cr^III^ together with its cheap character can be an alternative to luminescent supramolecular 4d-5d/4f materials^53,54^ and might open new avenues for the construction of luminescent cages or capsules with sensing and/or bioimaging applications.^55–57^ Thus, we report on what we believe to be the first synthesis and characterization of a tetrahedral Cr^III^_4_L_6_ (L = 2,2′:5′,5″:2″,2‴-quaterpyridine) cage displaying long-lived NIR luminescence.

## Results and Discussion

### Synthesis and Structural Analysis

The tetrahedral Cr^III^ cage, abbreviated as Cr^III^_4_L_6_ (L = 2,2′:5′,5″:2″,2‴-quaterpyridine), was prepared in a glovebox by reacting 4 equiv of the labile Cr^II^ (as CF_3_SO_3_ salt) with 6 equiv of L in dry acetonitrile. Subsequent oxidation of Cr^II^ to Cr^III^ was accomplished using a stoichiometric amount of silver triflate. After completion of the oxidation process, the mixture was filtered to remove the reduced metallic silver particles and concentrated under a vacuum. Diethyl ether was added to the concentrated solution, and an orange precipitate appeared, which was filtered and washed with dichloromethane and dried with diethyl ether. The solid was dissolved in water and filtered. A saturated solution of KPF_6_ was added obtaining an orange precipitate (see the SI for details). Slow diffusion of diethyl ether into a concentrated acetonitrile solution gave poor quality crystals after a few days but was suitable for single-crystal X-ray diffraction. The Cr^III^_4_L_6_ species crystallized in the rhombohedral space group *R*3̅*c* (Table S1). Similar to other reported tetrahedral cages, the four vertices are occupied by the Cr^III^ ions, which are linked to each other through coordinated bis-bidentate ligands (Figure 1). Each of the two bypiridyl (bipy) units of a given ditopic ligand is twisted with respect to the neighbor bipy unit by approximately 66°. The distance between each of the Cr^III^ centers is 9.76(3) Å, which corresponds to an encapsulated volume of approximately 99 Å^3^. Each of the vertices of the assembly is formed by a {Cr­(bipy)_3_}^3+^ moiety, with a typical Cr^III^–N bond length range of 1.95(4)–2.06(3) Å (Table S2) and bite angles of 80.7(12)° according to the five-membered chelate ring nature of the bipy scaffolds.^34^ The compound crystallized as a racemic mixture of the two homochiral ΔΔΔΔ and ΛΛΛΛ enantiomeric cages with a PF_6_^–^ anion located inside the cavity, a feature also observed in related cages (Figure 1b).^51^

**Figure 1:**
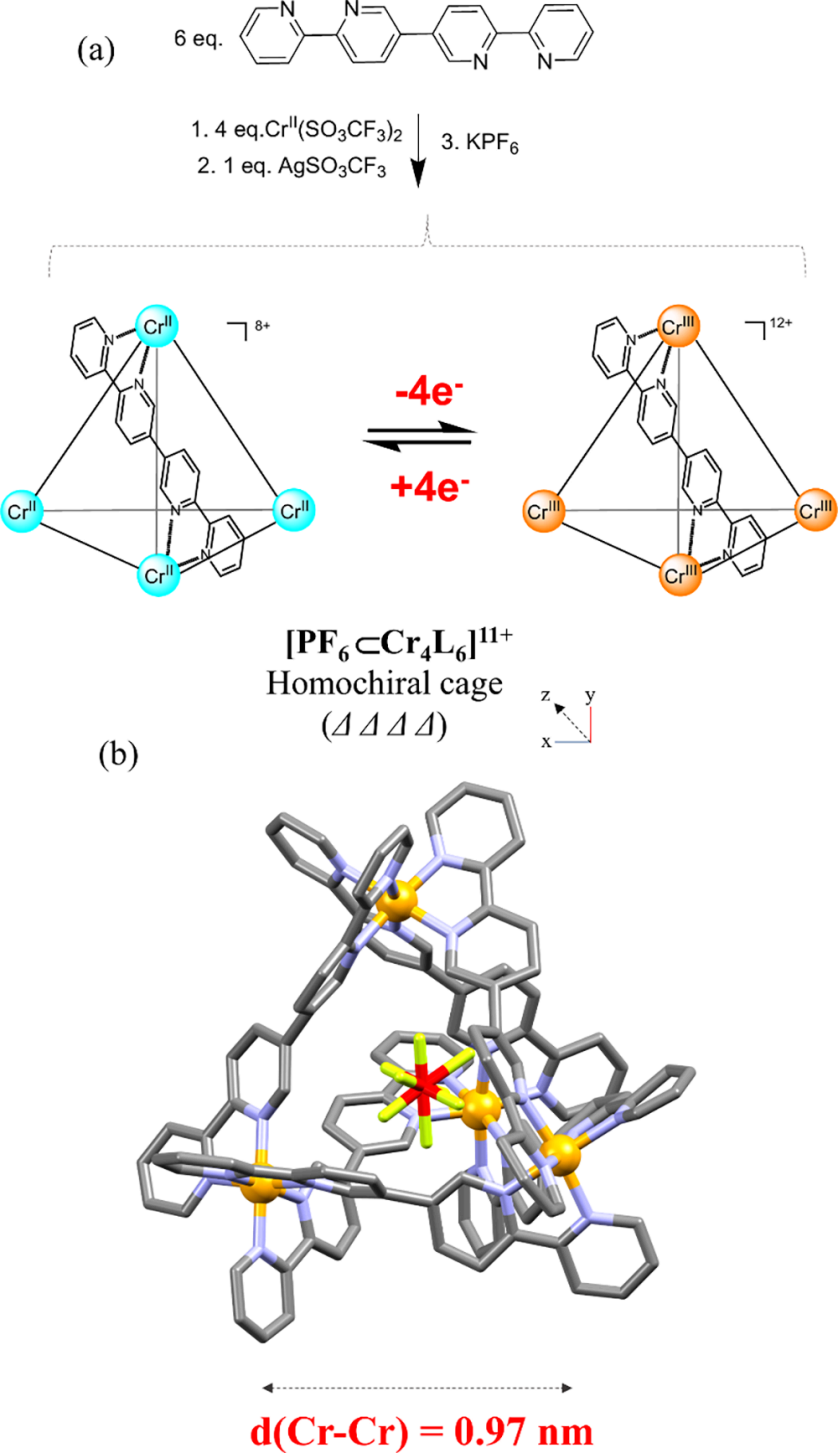
(a) Schematic synthetic pathway for the preparation of the Cr^III^_4_L_6_ cage. (b) X-ray molecular structure showing the ΔΔΔΔ enantiomer of the *rac*-Cr^III^_4_L_6_ encapsulating PF_6_^–^. The rest of the anions and the hydrogen atoms have been omitted for clarity. Color codes: Cr (orange), N (blue), C (gray), F (green), and P (red).

The ^19^F-NMR spectrum of the cage in CD_3_CN displayed broad features due to the paramagnetic and slow nuclear relaxation of Cr^III^ ions in its ground state (Figure S1).^58^ However, three signals located at −63 and −70 ppm and at −74 ppm can be distinguished (Figure S1b). The intense doublet corresponds to the noninteracting PF_6_ anions in the outer sphere of the cage, and the weaker one corresponds to strongly broadened doublet that corresponds to the PF_6_ anion inside the cavity. If the PF_6_^–^ anion would be in a single environment, we would expect to observe only one set of two signals due to the ^19^F–^31^P nuclei coupling (Figure S1a). In fact, it is reasonable to infer that these environments would correspond to endo and exo PF_6_^–^ anions, a feature also observed in similar cages.^49^ The high-resolution electrospray ionization mass spectrum (HR-ESI-MS) showed +5, +6 ions with masses corresponding to those calculated for successive losses of PF_6_^–^ anions from the parent species of formula Cr_4_L_6_(PF_6_)_12_ (Figure S2).

### Magnetic Properties

The dc magnetic properties of Cr^III^_4_L_6_ were studied in the 2–300 K temperature range with an applied magnetic field of 1000 Oe (Figure 2). The χ_M_ *T* value at room temperature of 7.75 cm^3^·mol^–1^·K matches that expected for four noninteracting Cr^III^ ions with *S* = 3/2 and *g* = 2 (7.75 cm^3^·mol^–1^·K). Upon cooling, the χ_M_ *T* product remains almost constant until about 20 K and then sharply decreases to reach a value of 6.15 K cm^3^·mol^–1^·K at 2 K. The decrease of χ_M_ *T* at a very low temperature is due to zero field splitting (ZFS) single-ion anisotropy and eventually weak intra- and intramolecular antiferromagnetic interactions. The field dependence of the magnetization in the 2–6 K range is given in the inset of Figure 2. The isotherm at 2 K is almost saturated at the maximum applied field of 7 T, reaching a value of 11.94 μ_B_ (*M*_sat_ = 12 μ_B_ for *S* = 3/2 and *g* = 2). The magnetic data were analyzed by the phenomenological approach based on the ZFS of an *S* = 3/2 through the following anisotropic spin-Hamiltonian:

$$Ĥ=-J_{ij}\sum_{i=1,J>i}^{4}S_{i}S_{j}+\sum_{i=1}^{i=4}D_{i}[{\hat{S}}_{zi}^{2}-S(S+1)/3]+\mu_{B}\sum_{i=1}^{i=4}\vec{g_{i}H_{i}}{\hat{S}}_{i}$$ 1

where the first term represents the intramolecular magnetic exchange interactions between the Cr^III^ ions and the second and third terms correspond to the single ion axial magnetic anisotropy and the Zeeman interaction, respectively. *J*_*ij*_ represents the corresponding magnetic coupling constants, *D* is the axial magnetic anisotropy parameter, μ_B_ is the Bohr magneton, and *H* is the applied magnetic field.

**Figure 2:**
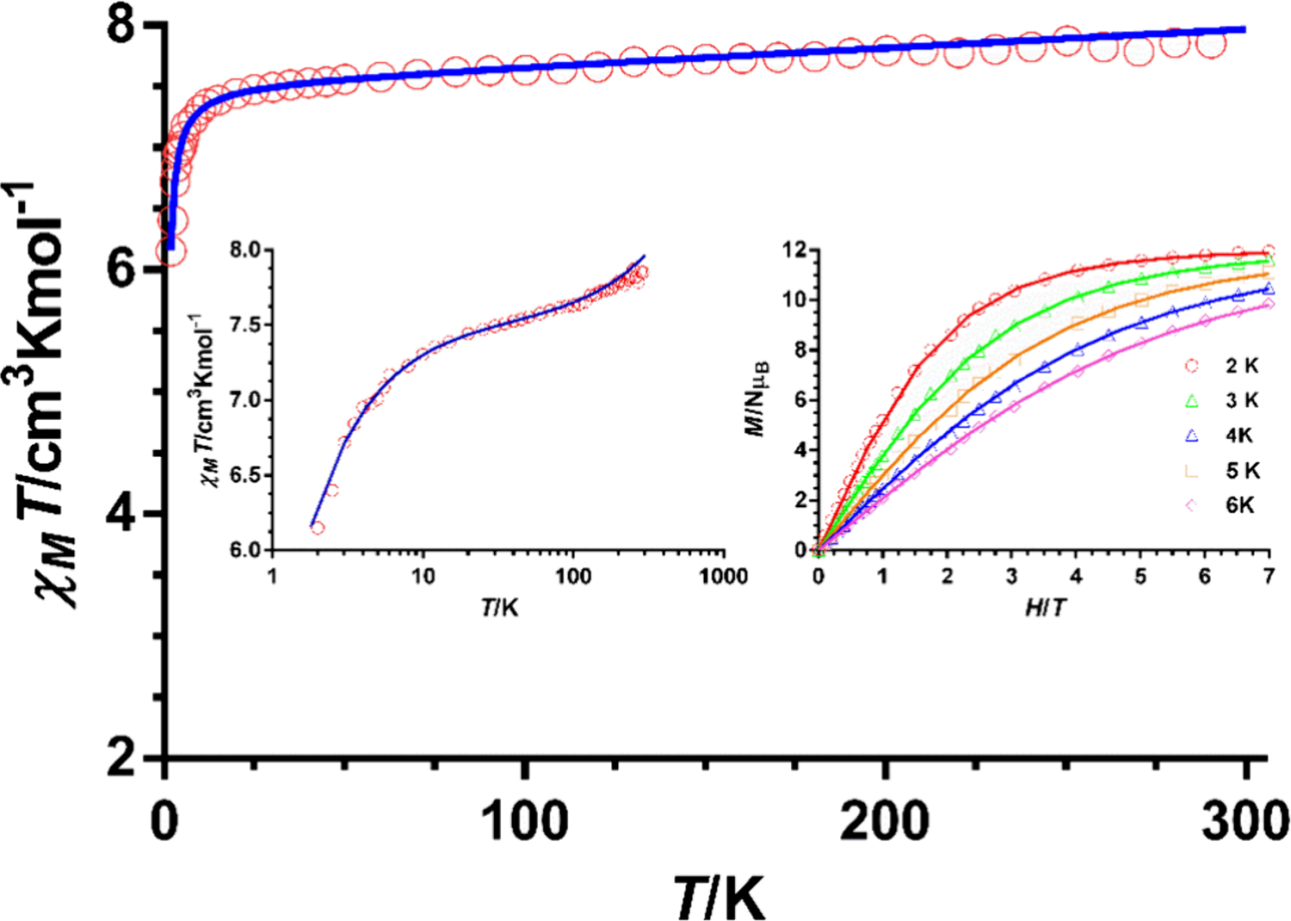
Temperature dependence of χ_M_ *T* (experimental: red circles; fit: blue line). Inset: χ_M_ *T* vs log *T* scale for highlighting the low temperature region (left) and field dependence of the magnetization at the indicated temperatures (inset). Solid lines represent the best fit to equation.

The susceptibility and magnetization data were simultaneously fitted using the PHI software^59^ to the above Hamiltonian. To avoid overparameterization, all the *J* coupling constants and all the local *D* values were considered equal. Moreover, a term accounting for temperature-independent paramagnetism (TIP) was also included in the Hamiltonian. A very good quality fit was obtained with the following parameters: *J* = −0.058(7) cm^–1^, *g* = 2.003(1), |*D*| = 0.58(3) cm^–1^, and TIP = 0.0015 cm^3^ mol^–1^. The extracted parameters confirm that the magnetic exchange interaction between the Cr^III^ ions is antiferromagnetic in nature and is very weak in magnitude. It should be mentioned that the low sensitivity of the powder magnetic measurements does not allow the accurate determination of the sign of the *D* parameter. However, the value remains in line with other Cr^III^ complexes (<1 cm^–1^).^60–67^ When either *J* or *D* were fixed to zero, the fits led to the following magnetic parameters: *g* = 1.990 (2), |*D*| = 1.14 (2) cm^–1^, and TIP = 0.0016 (1) cm^3^ mol^–1^ and *g* = 2.006 (1), *J* = −0.072 (1) cm^–1^, and TIP = 0.0012 (1) cm^3^ mol^–1^. Therefore, the *J* and *D* parameters are correlated and cannot be accurately determined from the magnetic data. Nevertheless, the values of *J* or *D* extracted when *D* or *J* was, respectively, fixed to zero can be considered as limits for these parameters. It is worth noting that the quality of the fit with *D* = 0 was slightly worse than that obtained when both *J* and *D* were allowed to freely vary. However, the fit with *J* = 0 is significantly worse than the other two fits, particularly below 10 K, thus indicating that *J* plays an essential role in the magnetic behavior of Cr^III^_4_L_6_. Although the dipolar magnetic interaction between metal ions with very small *D* values, like Cr^III^, is expected to be very weak, one might wonder if the very feeble magnetic coupling observed for Cr^III^_4_L_6_ could be dipolar in origin. To answer this question, we have estimated a value of the maximum dipolar contribution to the antiferromagnetic interaction in Cr^III^_4_L_6_ (when the magnetic anisotropic axes of two neighboring metal ions are parallel and the anisotropic axis and the Cr–Cr direction form an angle of 90°) of *J*_dip_ = 0.002 cm^–1^ (see the SI). This value is an order of magnitude smaller than the experimental *J* value, and therefore, the antiferromagnetic interaction is mainly due to magnetic exchange. Experimental magneto-structural studies on dinuclear Cr^III^ complexes with bis­(terpyridine)-type bridging ligands^64^ seem to support this conclusion because the complex [(tpy)­Cr­(bbt)­Cr­(tpy)]­(PF_6_)_6_·8CH_3_CN·(CH_3_CH_2_)_2_O (where tpy = 2,2′:6′,2″-terpyridine and bbt = 4,4‴-bis­(2,2′:6′,2″-terpyridine) with a Cr···Cr distance of 10.9 Å presents a magnetic coupling constant (*J* = −2.37 cm^–1^) smaller than that found for the complex ([(tpy)­Cr­(ebbt)­Cr­(tpy)]­(PF_6_)_6_·10 CH_3_CN (ebbt = 4,4‴-(ethynyl)-bis­(2,2′:6′,2″-terpyridine) of *J* = −2.73 cm^–1^) with a Cr···Cr distance of 13.5 Å. Therefore, the dipolar contribution to the magnetic coupling should be comparatively irrelevant in these types of complexes.

The magnetic exchange propagates across the π-system of the bridging ligand via overlap of its p­(π) orbitals and the t_2g_(π) orbitals of Cr^III^ ions, probably also involving a spin-polarization mechanism. In such a mechanism, the nitrogen donor atoms hold the same spin density as the Cr^III^ ions due to the spin delocalization in the Cr–N bonds, whereas the carbon atoms of the aromatic spacer possess an alternating sign of spin density owing to spin polarization, leading to the opposite sign of spin density on the Cr^III^ ions (antiferromagnetic interaction) when, like in this case, there is an even number of aromatic carbon atoms involving the magnetic exchange pathway.^68,69^ The very weak antiferromagnetic coupling found in the cage is a consequence of the long intramolecular distance between the Cr^III^ ions (9.76(3) Å) and the dihedral angle between the bipyridine units of the bridging ligand (twist angle). It is worth noting that the extracted *J* value is of the same order of magnitude as that previously reported for the Ni_4_ counterpart.^51^ The *J* value for this latter compound is larger than that found for the Cr^III^_4_L_6_ cage, which is mainly due to the higher number of unpaired electrons and the larger twist angle for the Cr^III^ counterpart (66° vs 44°). The fact that the *J* value for the dinuclear complex [(tpy)­Cr­(bbt)­Cr­(tpy)]­(PF_6_)_6_·8CH_3_CN·(CH_3_CH_2_)_2_O is much larger than that observed for the Cr^III^_4_L_6_ cage (−2.37 cm^–1^ vs −0.058 cm^–1^), even though the Cr···Cr distance and the twist angle are very close for both compounds (around 10 Å and 60°), could be due to the larger aromaticity of the terpyridine moiety. A more extended delocalized π-system lowers the energy of the LUMO and increases the π-back-donation from the metal to this orbital, and the ligand becomes a better π acceptor. This leads to a decrease in energy of the Cr-centered t_2g_ magnetic orbitals, thus favoring their overlap with the occupied molecular orbital of the bis­(terpyridine) bridging ligand and consequently the increase in the antiferromagnetic interaction. A similar increase in the antiferromagnetic magnetic interaction has been observed for bis­(terpyridine) bridged complexes when end-cap anionic π-donor chloride ligands are replaced by π-acceptor terpyridine ligands.^64^

### Absorption and Emission Properties

The absorption spectrum of the Cr^III^ cage in acetonitrile shows maxima between 300 and 350 nm (ε > 140 × 10^3^ M^–1^·cm^–1^), which can be attributed to standard intense π* ← π transitions, together with bands of lower intensities between 300 and 350 nm with (ε < 10^3^ M^–1^cm^–1^) assigned to mixed metal-centered and ligand-to-metal charge transfer MC/LMCT transitions (Figure 3). The absorption spectrum of the cage is slightly red-shifted compared to that of the mononuclear Cr^III^ compound, likely due to a bathochromic effect from the partially extended conjugation of the quarterpyridine ligands.

**Figure 3:**
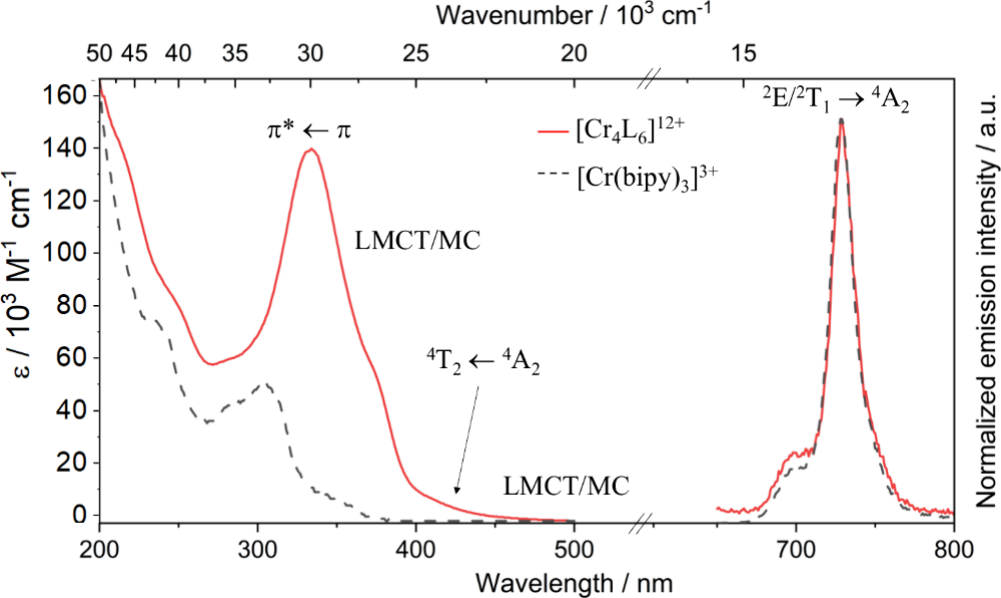
Absorption spectrum and emission spectrum of [Cr^III^_4_L_6_]^12+^ (red solid traces) and absorption spectrum and emission spectrum of [Cr­(bipy)_3_]^3+^ (dashed black traces) upon excitation at 330 nm at room temperature in acetonitrile with their respective band assignation.

The total ε values of the cage complex are around four times more important than those of mononuclear [Cr­(bipy)_3_]^3+^ due to the presence of four [Cr­(bipy)_3_]^3+^ units per molecular entity. Additionally, a similar band shape is observed in the whole spectra for both compounds. The weak transitions located at even lower energies (450–500 nm) are ascribed to the spin-forbidden ^3^π* ← π transitions.^28,31^ According to TD-DFT calculations on the model [Cr­(bipy)_3_]^3+^, the bands located between 400 and 430 nm can be attributed to the spin-allowed Cr­(^4^T_2_ ← ^4^A_2_) transition (Figures S4, S6, and S7 and Table S5). At lower energies, in the spin-flip region, no spin-forbidden ^2^E ← ^4^A_2_ and ^2^T_1_ ← ^4^A_2_ transitions could be identified even by using highly concentrated solutions. This may be attributed to the band tail of the LMCT, which obscures the spin-flip absorptions. At room temperature and upon excitation at 330 nm, the Cr^III^ cage exhibits a sharp emission centered at 727 nm (13755 cm^–1^) together with a small band located at 697 nm (14347 cm^–1^). The emission profile of the cage is identical to that of mononuclear [Cr­(bipy)_3_]^3+^, suggesting that both systems share the same low-lying excited state landscape (Figure 3). This similarity indicates that the coordination environment and electronic structure of the chromium centers in both the cage and the mononuclear complexes are similar, leading to indistinguishable emission spectra. This implies that any differences in the physical arrangement or macroscopic properties of the cage do not affect the electronic excited states of the chromium centers. According to theoretical calculations on the mononuclear complex (CASSCF­(7,12)/FIC-NEVPT2), the energies of the lowest ^2^E and ^2^T_1_ states are similar and suggest that the lowest energy emission band could be attributed to a ^2^T_1_ → ^4^A_2_ transition (Tables S3 and S4 Figure S5). The luminescence quantum yield (ϕ) of the Cr^III^ cage and [Cr­(bipy)_3_]^3+^ in CH_3_CN was determined by a relative method using [Cr­(ddpd)_2_]^3+^ (*N,N’* dimethyl-*N,N’*-dipyridine-2-yl-pyridine-2,6-diamine) as reference (see the SI for details).^16^ The value obtained in aerated conditions is 0.044%, while in deaerated conditions, it increases to 0.067%. In both cases, these values are slightly higher compared to the mononuclear [Cr­(bipy)_3_]^3+^ (ϕ = 0.030% (aerated), ϕ = 0.057% (deaerated)). Similarly, the excited state lifetime of the Cr^III^ cage shows a monoexponential decay with a value of τ = 79 μs (aerated) and increases almost twice to reach τ = 183 μs in a deaerated solution (Figure S3). In the case of [Cr­(bipy)_3_]^3+^, this value increases from 40 (aerated) to 102 μs (deaerated) (Figure S3). The longer excited-state lifetime observed in the metallosupramolecular species is likely attributed to increased rigidity, which hinders certain nonradiative relaxation pathways. Finally, the presence of four [Cr­(bipy)_3_]^3+^ units in the cage together with the little increase in the luminescence quantum yield led to an enhancement of the photoluminescence brightness, *B* (determined as *B* = ε·ϕ), that goes from 14 M^–1^·cm^–1^ for the mononuclear complex to 90 M^–1^·cm^–1^ for the cage, calculated by excitation at λ_abs,max_ for each complex. This represents nearly a 6-fold improvement. Thus, increasing the number of chromophores within the same molecular structure can be a remedy for increasing the photoluminescence performance of poor spin-flip emitters such as the archetypal [Cr­(bipy)_3_]^3+^ complex.

## Conclusions

The first tetrahedral Cr^III^ cage was prepared using an “assembly followed by oxidation” approach. Initially, labile Cr^II^ reacts with the appropriate amount of ligand, which is subsequently oxidized to produce the kinetically inert [Cr_4_^III^L_6_]^12+^ cage, which encapsulates a PF_6_^–^ anion as confirmed by single-crystal X-ray diffraction. The four Cr^III^ ions located at the corners of the tetrahedron exhibit antiferromagnetic exchange interactions at low temperatures. Upon light excitation, spin-flip emission from the radiative relaxation of doublet excited states ^2^E/^2^T_1_ to ground state ^4^A_2_ was detected at room temperature. Both the luminescence quantum yield and the excited-state lifetime under aerobic and anaerobic conditions at room temperature are slightly higher compared with the mononuclear analogue [Cr­(bipy)_3_]^3+^. This improvement is attributed to the more rigid structure of the assembled cage, which prevents structurally related nonradiative relaxation pathways. Additionally, the presence of up to four [Cr­(bipy)_3_]^3+^ units in the cage significantly enhances the photoluminescence brightness of the cage compared to the mononuclear complex.

## Experimental Part

″No uncommon hazards are noted.″

Elemental analyses were carried out on a Fisons-Carlo Erba analyzer model EA 1108. ^1^H nuclear magnetic resonance spectroscopy (RMN) data were recorded on a 400 MHz BRUKER Nanobay Avance III HD High-Definition spectrometer, and the spectra were internally referenced to solvent signals. Absorption spectra in acetonitrile solution were recorded using a Jasco Cary (Agilent Technologies) spectrometer (quartz cell path length 1 cm or 1 mm, 290–800 nm domain, 2 × 10^–4^ M and 650–800 nm domain, 7.7 mM). Emission and excitation spectra were measured on a UV–vis-PTI QuantaMaster 8000 spectrofluorometer equipped with a Picosecond Photon Detector (230–850 nm, PPD-850, HORIBA Scientific) and a continuous Xenon Short Arc Lamp (190–2000 nm, USHIO). All of the spectra (emission and excitation) were corrected with real-time correction functions. TCSPC lifetime measurements were performed using a flash lamp (1 μs pulse, HORIBA Scientific). All emission quantum yields were measured according to a relative method using the [Cr­(ddpd)_2_]^3+^ as reference (λ_exc_ = 435 nm; ϕ = 11% in deaerated acetonitrile; estimated uncertainty ±10%). Deaerated solutions were prepared in a 1 cm cuvette inside a glovebox filled with Ar. Variable-temperature (2–300 K) magnetic susceptibility measurements were carried out on polycrystalline samples under an applied field of 1000 Oe using DynaCool PPMS-9 physical measurement equipment. High-resolution ESIMS of the cage was recorded on a ThermoFischer Exactive Orbitrap using acetonitrile as the solvent.

## X-ray Crystallography

Single-crystal X-ray diffraction studies were performed on a Bruker D8 VENTURE equipped with a Photon 3 detector and graphite monochromated Mo Kα radiation (λ = 0.71073 Å) diffractometer. The temperature during data collection was controlled by means of a dry N_2_(g) cryostream (Oxford Cryostream 800). All data was collected using Mo Kα (λ = 0.711 Å) radiation. Suitable single crystals were selected and mounted on MiTeGen polymer loops. APEX 3 software was used to collect and reduce the data. Adsorption corrections were applied using empirical methods using symmetry equivalent reflections combined with measurements at different azimuthal angles implemented using SABADS. The structure was initially solved using the SHELXT (Supporting Information, S1) program using an intrinsic phasing method implemented through OLEX 2 (Supporting Information, S2) (v1.5) and refined using SHELXL (Supporting Information, S3) least-squares refinement procedures against all *F* ^2^ values. The solvent was particularly disordered, and the solvent mask protocol implemented in the OLEX 2 procedure was applied to estimate the electrons within the void space, accounting with a PF_6_^–^ anion and 4.5 molecules of acetonitrile. All nonmetal atoms were refined isotropically to maximize the data/parameter ratio. Hydrogen atoms were placed in calculated positions and refined with idealized geometries and assigned fixed occupancies and isotropic displacement parameters. PF_6_^–^ molecules were located after completion of the framework structure by inspection of residual electron density and were modeled by fitting a rigid body model to electron density. X-ray diffraction data for crystals of [Cr^III^_4_L_6_]­(PF_6_)_12_ presented a resolution of 1.35 Å, so the data was trimmed accordingly. This resolution is typical of these cages, and traditionally synchrotron X-ray source is used to elucidate the structure of the macromolecules (Supporting Information, S4 and S5). The high R1 and wR2 values are a consequence of the poor resolution, which intrinsically produced ill-shaped electron density maps. Fitting and refine of the atomic independent model built based on the ill-shaped electron density produce unusually high R1 and wR2 values. Crystallographic information files CCDC 2368147 contain full details for the crystal structure reported.

## Theoretical Studies

The Orca ((Supporting Information, S6)­(version 5.0.4) software package was used to investigate the structural and electronic properties of [Cr­(bipy)_3_]^3+^. Starting from the X-ray diffraction structures, the ground, ^4^A_2_ state, structure of [Cr­(bipy)_3_]^3+^ was obtained from DFT optimizations using the unrestricted version of the Becke three-parameter exchange function in combination with the Lee–Yang–Parr correlation functional (UB3LYP). The Ahlrichs’ polarized valence triple-ζ basis set def2-TZVPP was used for these optimizations. The D3 version of Grimme’s dispersion with Becke–Johnson damping (GD3BJ) was applied. Solvent effects were included via the conductor-like polarizable continuum model (CPCM) as implemented in Orca 5.0.4 with the dielectric constant of acetonitrile. Optimized geometries were confirmed to be stationary points by the analysis of their vibrational frequencies. Tight convergence criteria were selected for the optimization step. The resolution of identity approach for the Coulomb term in combination with the chain-of-spheres approximation for the exchange term (RIJCOSX) was applied. The zero-order relativistic approximation (ZORA) was used to describe relativistic effects for [Cr­(bipy)_3_]^3+^. Spin density information was extracted from the optimized geometries.

To accurately model the ligand field, the complete-active-space self-consistent field method (CASSCF) together with the fully internally contracted N-electron valence perturbation theory to second order (FIC-NEVPT2) was used. Dominant bonding/antibonding orbitals formed between the ligand and chromium and a second d shell were considered, creating an active space of 7 electrons and 12 orbitals (CASSCF­(7,12)/FIC-NEVPT2). Ten quartet and nine doublet roots were computed to calculate the energies of the excited states.

The 100 lowest energetic transitions were calculated by TD-DFT as implemented in Orca 5.0.4, using the unrestricted version of the B3LYP functional, selecting the def2-TZVPP basis set, and considering solvent effects. Electronic transitions were corrected by −0.2 eV to better fit the experimental results.

## Supplementary Material



## Notes

Complete experimental details, complex syntheses, characterizations, and photophysical data (DOCX)
