# Supplementary material for: Self-Assembled Tetrahedral [CrIII 4L6]12+ Cage Displaying Near-Infrared Spin-Flip Photoluminescence
Source: Inorg Chem. 2024 Nov 28;63(50):23886–93. doi: 10.1021/acs.inorgchem.4c04180 (PMC12305491; doi:10.1021/acs.inorgchem.4c04180)
Supplement: Supplementary file 1 [file ic4c04180_si_001.docx]

SUPPORTING INFORMATION

Self-Assembled Tetrahedral [Cr^III^_4_L_6_]^12+^ Cage Displaying Near-Infrared Spin-Flip photoluminescence

Yating Ye, ^†^ Carlos M. Cruz, ^‡^ Benjamin Doistau, ^§^ Enrique Colacio, ^†^ Claude Piguet, ^||^ Juan Manuel Herrera, ^†^ Juan-Ramón Jiménez^†*^

^†^Department of Inorganic Chemistry. University of Granada and “Unidad de Excelencia en Química (UEQ)”, Avda. Fuente Nueva s/n, 18071, Granada, Spain.

^‡^ Department of Organic Chemistry. University of Granada and “Unidad de Excelencia en Química (UEQ)”, Avda. Fuente Nueva s/n, 18071, Granada, Spain.

^§^Laboratoire de Chimie et de Biochimie Pharmacologiques et Toxicologiques (UMR 8601), Université Paris Cité, CNRS, 45 rue des Saint-Pères F-75006 Paris, France.

^||^Department of Inorganic and Analytical Chemistry, University of Geneva, 30 quai E. Ansermet, CH-1211 Geneva 4, Switzerland.

**Supporting Information**

(22 pages)

**Solvents and starting materials**

All the chemicals were purchased from commercial suppliers and used without further purification.

**Synthesis**

**Synthesis of 2,2*’*:5*’*,5*’’*:2*’’*,2*’’’*-quaterpyridine (L).**

NiCl_2_ (1,16 g, 8,96 mmol) and PPh_3_ (0,88 g, 3,36 mmol) were added in a Schlenk tube and vacuum and N_2_ cycles were performed several times. Anhydrous DMF (10 mL) was added, and the solution became dark blue upon heating at 50ºC for 2 h, once cooled to room temperature, Zn dust (0,6 g, 9,17 mmol) was added, and after approximately 2 h, the solution turned dark green. A solution of 10 mL of anhydrous DMF containing Br(bpy) (2g, 8,5 mmol) was then added, and the mixture was stirred at room temperature for approximately 16 h. Subsequently, the solvent was removed under vacuum, and an EDTA (31,64 g, 85 mmol)/NaOH (6,8 g, 170 mmol) solution (200 mL) and CH_2_Cl_2_ (100 mL) was added, the mixture was stirred at room temperature for 24 h. At the end of the reaction, the solution became pale blue. After separating the organic phase, the aqueous phase was extracted using CH_2_Cl_2_ three times. The combined organic phases were dried over MgSO_4_, filtered, and evaporated under vacuum, the yellow precipitates were formed, vacuum filtered and dried with Et_2_O. Yield: 1 g (76 %). ^1^H NMR (400 MHz, CDCl_3_) δ (ppm): 8.99 (d, *J* = 2.4 Hz, 2H), 8.75 – 8.69 (m, 2H), 8.55 (d, *J* = 8.3 Hz, 2H), 8.47 (d, *J* = 8.0 Hz, 2H), 8.10 (dd, *J* = 8.2, 2.4 Hz, 2H,), 7.86 (td, *J* = 7.7, 1.8 Hz, 2H,), 7.34 (dd, *J* = 7.5, 4.9 Hz, 2H,). ESI-MS (*m/z*): 311 [L+H]^+^. Elemental analysis calculated for C_20_H_14_N_4_ · 0.3 H_2_O: C, 76.08 %; H, 4.66 %; N, 17.74 %. Found: C, 76.14 %; H, 4.72 %; N, 17.59 %.

**Synthesis of the cage [Cr^III^_4_L_6_](PF_6_)_12_.**

**Scheme S1.** Schematic representation of the synthesis of tetrahedral Cr^III^ cages.

In the glovebox, ligand L (121 mg, 0,39 mmol) was suspended in anhydrous CH_3_CN (10 mL) and added dropwise to a solution containing Cr(SO_3_CF_3_)_2_ · 2H_2_O (100 mg, 0,26 mmol) previously dissolved in 5 mL of anhydrous CH_3_CN, resulting in a purple solution. After 3 h of stirring, AgCF_3_SO_3_ (67 mg, 0,26 mmol) was added, leading to a colour change from purple to orange. The mixture was stirred again for 3 h, during which a grey solid appeared. Upon this period, the solution was removed from the glovebox and filtered through a PTFE membrane of 0,22 $\mu$m. The volume of the orange solution was reduced under vacuum and Et_2_O was added to precipitate the solids, which were filtered. The obtained orange solid was then dissolved in H_2_O (15 mL) and drops of a saturated aqueous solution of KPF_6_ were added. The orange precipitate was filtered, washed with cold CH_2_Cl_2_, Et_2_O and dried under vacuum. Single crystals suitable for XDR were obtained by slow diffusion of Et_2_O into a concentrated CH_3_CN solution. Yield: 100 mg (40 %). Elemental analysis calculated for C_120_H_84_Cr_4_F_72_N_24_P_12_ · 3.7 H_2_O: C, 37.18 %; H, 2.38 %; N, 8.67 %. Found: C, 37.66 %; H, 2.86 %; N, 8.33 %.

**Table S1.** Crystal data and structure refinement for [Cr^III^_4_L_6_](PF_6_)_12_

| CCDC number | 2368147 |
| --- | --- |
| Empirical formula | C_129_H_97.5_Cr_4_F_72_N_28.5_P_12_ |
| Formula weight | 3994.49 |
| Temperature/K | 150.00 |
| Crystal system | trigonal |
| Space group | R-3c |
| a/Å | 18.930(4) |
| b/Å | 18.930(4) |
| c/Å | 167.03(5) |
| α/° | 90 |
| β/° | 90 |
| γ/° | 120 |
| Volume/Å^3^ | 51832(26) |
| Z | 12 |
| ρ_calc_g/cm^3^ | 1.536 |
| μ/mm^‑1^ | 0.487 |
| F(000) | 23940.0 |
| Crystal size/mm^3^ | 0.11 × 0.1 × 0.03 |
| Radiation | MoKα (λ = 0.71073) |
| 2Θ range for data collection/° | 4.222 to 30.516 |
| Index ranges | -13 ≤ h ≤ 13, -13 ≤ k ≤ 14, -123 ≤ l ≤ 123 |
| Reflections collected | 44287 |
| Independent reflections | 2455 [R_int_ = 0.1939, R_sigma_ = 0.0600] |
| Data/restraints/parameters | 2455/477/261 |
| Goodness-of-fit on F^2^ | 2.042 |
| Final R indexes [I>=2σ (I)] | R_1_ = 0.1928, wR_2_ = 0.4719 |
| Final R indexes [all data] | R_1_ = 0.2274, wR_2_ = 0.4970 |
| Largest diff. peak/hole / e Å^-3^ | 1.57/-0.68 |

**ORTEP view**


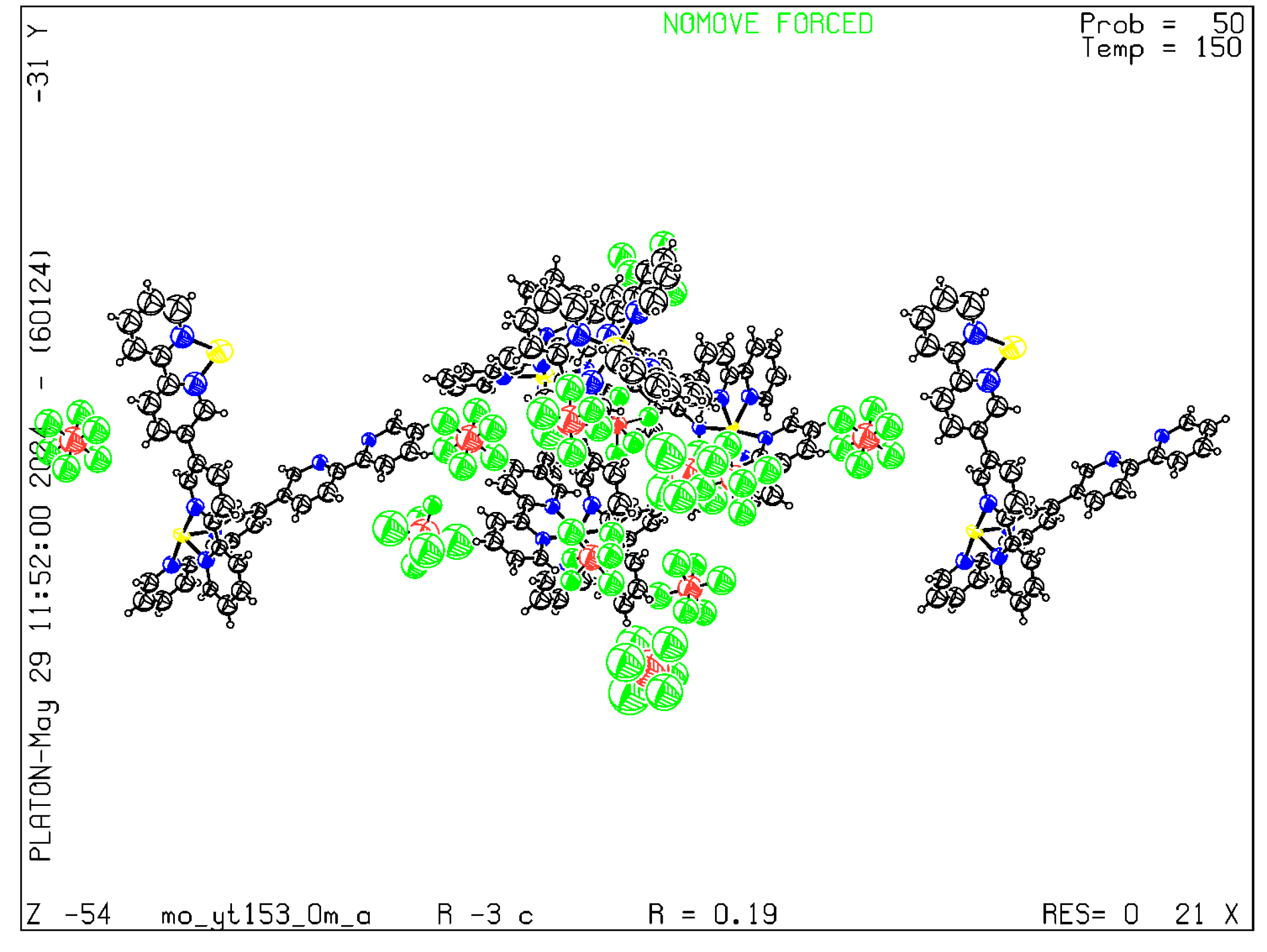


| **Table S2. Bond Lengths for [Cr^III^_4_L_6_](PF_6_)_12_** | | | | | | |
| --- | --- | --- | --- | --- | --- | --- |
| **Atom** | **Atom** | **Length/Å** |  | **Atom** | **Atom** | **Length/Å** |
| Cr(1) | N(2) | 2.00(3) |  | C(37) | C(38) | 1.34(4) |
| Cr(1) | N(4) | 2.06(3) |  | C(39) | C(38) | 1.47(4) |
| Cr(1) | N(3) | 2.02(3) |  | C(28) | C(27) | 1.44(5) |
| Cr(1) | N(1) | 2.008(18) |  | C(21) | N(5) | 1.3900(12) |
| Cr(1) | N(7)^1^ | 2.01(9) |  | C(21) | C(22) | 1.3900 |
| Cr(1) | N(8)^1^ | 2.02(15) |  | N(5) | C(25) | 1.3900 |
| Cr(2) | N(6)^1^ | 1.95(4) |  | C(25) | C(24) | 1.3900 |
| Cr(2) | N(6)^2^ | 1.95(4) |  | C(24) | C(23) | 1.3900(14) |
| Cr(2) | N(6) | 1.95(4) |  | C(23) | C(22) | 1.3900 |
| Cr(2) | N(5)^2^ | 2.04(4) |  | P(1) | F(1) | 1.6478 |
| Cr(2) | N(5) | 2.04(2) |  | P(1) | F(2) | 1.6390 |
| Cr(2) | N(5)^1^ | 2.04(6) |  | P(1) | F(9) | 1.6455 |
| N(2) | C(6) | 1.37(3) |  | P(1) | F(10) | 1.6542 |
| N(2) | C(10) | 1.35(3) |  | P(1) | F(11) | 1.6474 |
| N(4) | C(32) | 1.32(4) |  | P(1) | F(12) | 1.6457 |
| N(4) | C(35) | 1.31(4) |  | P(2) | F(3) | 1.6468 |
| N(3) | C(36) | 1.34(4) |  | P(2) | F(4) | 1.6466(17) |
| N(3) | C(40) | 1.43(4) |  | P(2) | F(5) | 1.6457 |
| N(1) | C(5) | 1.3900 |  | P(2) | F(6) | 1.641(2) |
| N(1) | C(1) | 1.3900 |  | P(2) | F(7) | 1.6479(19) |
| C(5) | C(4) | 1.3900 |  | P(2) | F(8) | 1.643(2) |
| C(5) | C(6) | 1.42(3) |  | P(3) | F(13) | 1.6482 |
| C(4) | C(3) | 1.3900 |  | P(3) | F(14) | 1.6424 |
| C(3) | C(2) | 1.3900 |  | P(3) | F(15) | 1.6444(12) |
| C(2) | C(1) | 1.3900 |  | P(3) | F(16) | 1.6502(11) |
| C(6) | C(7) | 1.37(4) |  | P(3) | F(17) | 1.6448 |
| N(6) | C(30) | 1.36(5) |  | P(3) | F(18) | 1.6505 |
| N(6) | C(26) | 1.34(5) |  | P(4) | F(19) | 1.6467(19) |
| C(10) | C(9) | 1.33(4) |  | P(4) | F(1A) | 1.6500(12) |
| C(9) | C(12) | 1.47(3) |  | P(4) | F(1B) | 1.648(2) |
| C(9) | C(8) | 1.46(4) |  | P(4) | F(1C) | 1.6438 |
| C(15) | N(7) | 1.3900(16) |  | P(4) | F(1D) | 1.6471 |
| C(15) | C(14) | 1.3900 |  | P(4) | F(1E) | 1.6421 |
| C(15) | C(16) | 1.40(2) |  | P(5) | F(1F) | 1.6468 |
| N(7) | C(11) | 1.3900 |  | P(5) | F(1G) | 1.6444 |
| C(11) | C(12) | 1.3900 |  | P(5) | F(1H) | 1.6445 |
| C(12) | C(13) | 1.3900(15) |  | P(5) | F(1I) | 1.6475 |
| C(13) | C(14) | 1.3900 |  | P(5) | F(1J) | 1.646(3) |
| C(36) | C(35) | 1.31(4) |  | P(5) | F(1K) | 1.647(3) |
| C(36) | C(37) | 1.41(4) |  | P(6) | F(1L) | 1.646(7) |
| C(7) | C(8) | 1.34(4) |  | P(6) | F(1M) | 1.6507 |
| C(32) | C(31) | 1.36(4) |  | P(6) | F(1N) | 1.645(7) |
| C(40) | C(39) | 1.38(4) |  | P(6) | F(1O) | 1.6443 |
| C(29) | C(30) | 1.45(4) |  | P(6) | F(1P) | 1.6460(18) |
| C(29) | C(31) | 1.51(4) |  | P(6) | F(1Q) | 1.6450(17) |
| C(29) | C(28) | 1.33(4) |  | P(7) | F(1R) | 1.646(3) |
| C(35) | C(34) | 1.28(5) |  | P(7) | F(1S) | 1.6520(17) |
| C(26) | C(25) | 1.47(5) |  | P(7) | F(1T) | 1.645(4) |
| C(26) | C(27) | 1.31(5) |  | P(7) | F(1U) | 1.6428(19) |
| C(17) | C(18) | 1.3900 |  | P(7) | F(1V) | 1.647(3) |
| C(17) | C(16) | 1.3900(17) |  | P(7) | F(1W) | 1.646(2) |
| C(18) | C(19) | 1.3900 |  | P(9) | F(1X) | 1.6458 |
| C(19) | C(20) | 1.3900(14) |  | P(9) | F(1Y) | 1.646(5) |
| C(20) | N(8) | 1.3900 |  | P(9) | F(1Z) | 1.6447 |
| N(8) | C(16) | 1.3900 |  | P(9) | F(22) | 1.646(5) |
| C(31) | C(33) | 1.39(5) |  | P(9) | F(23) | 1.6492 |
| C(34) | C(33) | 1.51(5) |  | P(9) | F(24) | 1.6452 |

**Figure S1.** a) ^19^F-NMR of the KPF_6_ in D_2_O. b) ^19^F-NMR spectrum of [Cr^III^_4_L_6_](PF_6_)_12_ in acetonitrile-d_3_ at 300 K.

**
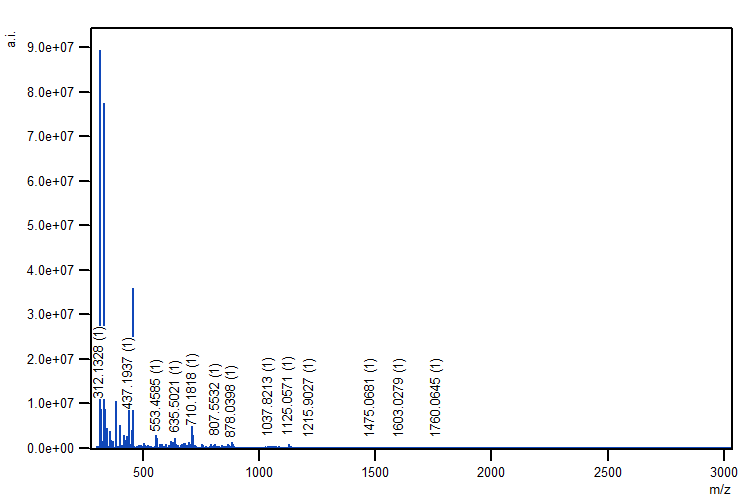
**

**Figure S2.** Top: full HR-MS. Down: Experimental and simulated mass spectra for the {Cr_4_L_6_(PF_6_)_6_}^6+^ and {Cr_4_L_6_(PF_6_)_7_}^5+^ in MeOH.

**Figure S3.** a) Excited-state decay curve (black points) and the best fit of the experimental data (red solid line) of the Cr(III) cage under Ar at r.t. b) of the [Cr(bipy)_3_]^3+^ under Ar at r.t. c) of the Cr(III) cage under O_2_ atmosphere at r.t. d) of the [Cr(bipy)_3_]^3+^ under O_2_ atmosphere at r.t.

*Optimized Geometries*


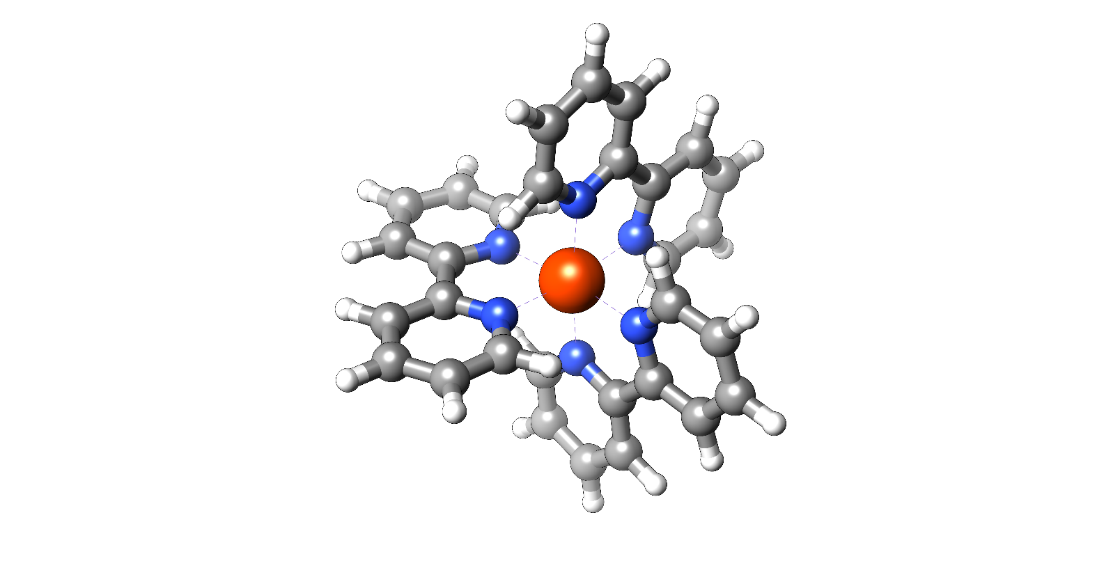


**Figure S4**. DFT optimized geometries of the quartet ground state of [Cr(bipy)_3_]^3+^. Spin density at the Cr center: 3.203347.

*Complete active space analysis of excited states*

**Table S3.** CASSCF(7,12)/FIC-NEVPT2 results [Cr(bipy)_3_]^3+^. Energies in cm^-1^.

|  | ^2^E (1) | ^2^E (2) | ^2^T_1_ (1) | ^2^T_1_ (2) | ^2^T_1_ (3) | ^2^T_2_ (1) | ^2^T_2_ (2) | ^2^T_2_ (3) | ^4^T_2_ (1) | ^4^T_2_ (2) | ^4^T_2_ (3) |
| --- | --- | --- | --- | --- | --- | --- | --- | --- | --- | --- | --- |
| [Cr(bipy)_3_]^3+^ | 15449 | 15694 | 15447 | 16111 | 16114 | 23223 | 23224 | 24031 | 23795 | 23948 | 23969 |

**Figure S5.** Schematic representation of the energy levels for the calculated (CASSCF(7,12)/FIC-NEVPT2) excited states of [Cr(bipy)_3_]^3+^.

**Table S4.** Orbitals used in the CASSCF(7,12)/FIC-NEVPT2 calculations for [Cr(bipy)_3_]^3+^.

| # | E (hartrees) | Orbital | # | Energy | Orbital |
| --- | --- | --- | --- | --- | --- |
| 130 | -0.888935 | 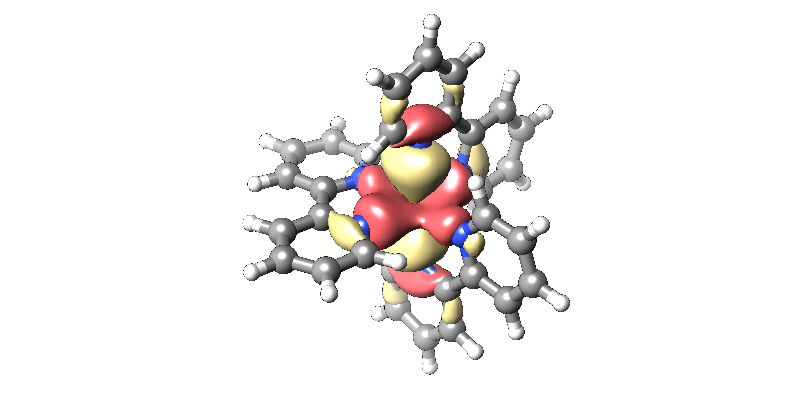 | 136 | -0.208100 | 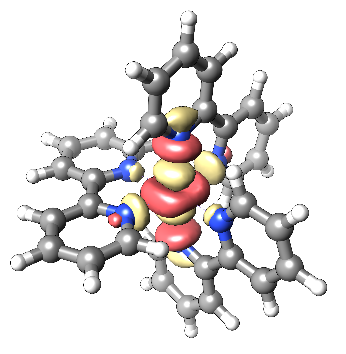 |
| 131 | -0.889076 | 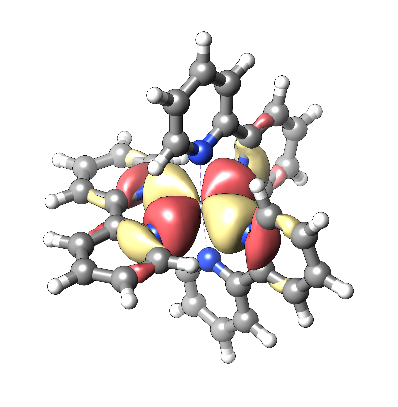 | 137 | 0.677293 | 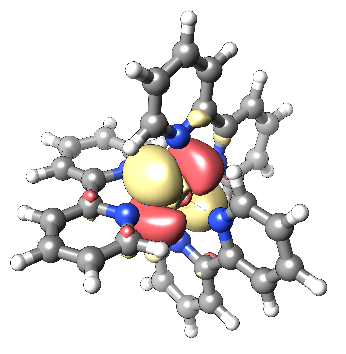 |
| 132 | -0.423948 | 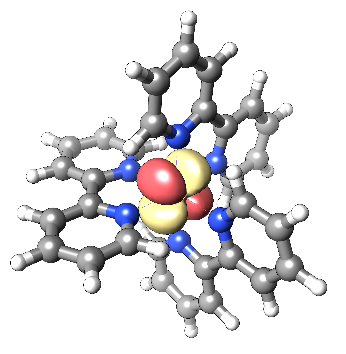 | 138 | 0.677798 | 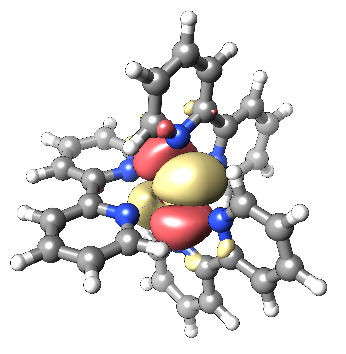 |
| 133 | -0.423935 | 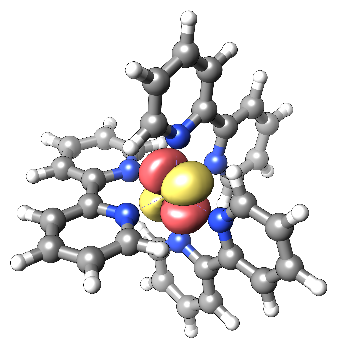 | 139 | 0.727715 | 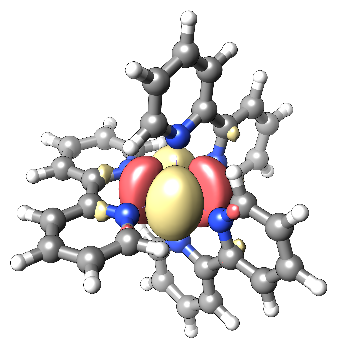 |
| 134 | -0.423964 | 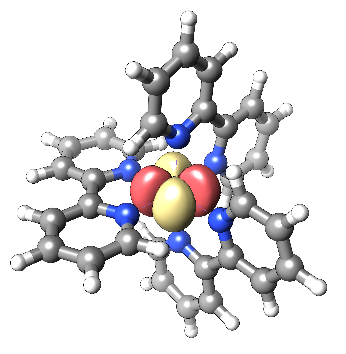 | 140 | 1.360899 | 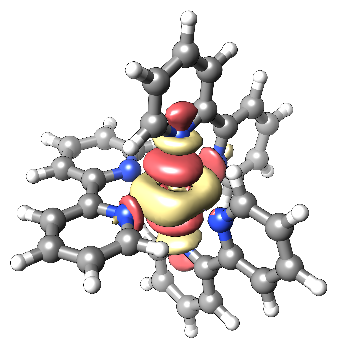 |
| 135 | -0.208340 | 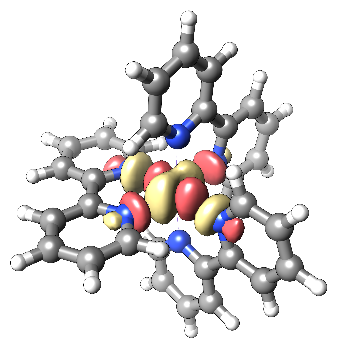 | 141 | 1.361887 | 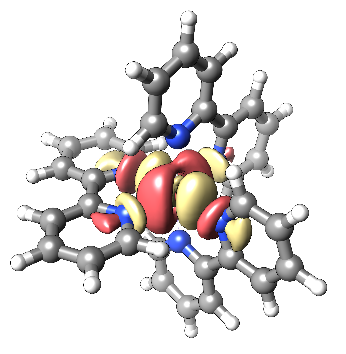 |

*Electronic transitions*

**Table S5.** Calculated 100 lowest electronic transitions for compound [Cr(bipy)_3_]^3+^, their energies (in nm) and oscillator strength (in cgs units). Correction: –0.2 eV

| Transition number | Wavelength / nm | Osc. Strength / cgs | Transition number | Wavelength / nm | Osc. Strength / cgs |
| --- | --- | --- | --- | --- | --- |
| 1 | 432.96 | 0.010072100 | 51 | 271.71 | 0.008333670 |
| 2 | 431.36 | 0.004127080 | 52 | 271.38 | 0.006354960 |
| 3 | 431.24 | 0.004128540 | 53 | 271.27 | 0.000044597 |
| 4 | 424.27 | 0.000041616 | 54 | 271.16 | 0.008936150 |
| 5 | 423.93 | 0.000006311 | 55 | 270.07 | 0.024237400 |
| 6 | 421.31 | 0.000310376 | 56 | 269.96 | 0.024875800 |
| 7 | 375.55 | 0.000000713 | 57 | 269.31 | 0.188820000 |
| 8 | 371.84 | 0.003801480 | 58 | 268.98 | 0.032286100 |
| 9 | 371.61 | 0.003862170 | 59 | 268.77 | 0.029119500 |
| 10 | 359.28 | 0.002642490 | 60 | 268.44 | 0.000000005 |
| 11 | 359.17 | 0.001917980 | 61 | 268.22 | 0.025315500 |
| 12 | 354.92 | 0.025071100 | 62 | 268.00 | 0.008266410 |
| 13 | 354.69 | 0.030707700 | 63 | 267.57 | 0.051983400 |
| 14 | 354.47 | 0.000003761 | 64 | 265.39 | 0.008011030 |
| 15 | 353.80 | 0.084178600 | 65 | 265.39 | 0.008162390 |
| 16 | 325.32 | 0.002114780 | 66 | 262.79 | 0.000003989 |
| 17 | 325.10 | 0.002326240 | 67 | 262.46 | 0.012092500 |
| 18 | 322.22 | 0.000001302 | 68 | 259.85 | 0.027051100 |
| 19 | 319.23 | 0.068941000 | 69 | 259.74 | 0.027312100 |
| 20 | 319.01 | 0.033806700 | 70 | 258.55 | 0.012600200 |
| 21 | 318.68 | 0.048239000 | 71 | 258.44 | 0.011924600 |
| 22 | 315.25 | 0.000008892 | 72 | 258.01 | 0.067814900 |
| 23 | 314.26 | 0.000062166 | 73 | 256.27 | 0.000101879 |
| 24 | 314.15 | 0.000000845 | 74 | 256.27 | 0.000102345 |
| 25 | 310.62 | 0.000000290 | 75 | 253.56 | 0.001126910 |
| 26 | 310.51 | 0.000010869 | 76 | 252.26 | 0.019691400 |
| 27 | 308.19 | 0.001394540 | 77 | 252.15 | 0.018505800 |
| 28 | 305.99 | 0.012860800 | 78 | 251.50 | 0.000003581 |
| 29 | 305.33 | 0.000604721 | 79 | 251.40 | 0.050554400 |
| 30 | 305.33 | 0.000311592 | 80 | 250.53 | 0.002721810 |
| 31 | 304.23 | 0.107643000 | 81 | 250.42 | 0.002813210 |
| 32 | 304.12 | 0.103656000 | 82 | 249.45 | 0.002308880 |
| 33 | 302.91 | 0.011578000 | 83 | 249.45 | 0.002669290 |
| 34 | 302.80 | 0.013832200 | 84 | 248.91 | 0.000026572 |
| 35 | 302.03 | 0.000000443 | 85 | 248.91 | 0.000375317 |
| 36 | 298.95 | 0.395044000 | 86 | 248.47 | 0.000007664 |
| 37 | 294.23 | 0.005901130 | 87 | 247.83 | 0.008309640 |
| 38 | 294.23 | 0.004164030 | 88 | 247.61 | 0.003231480 |
| 39 | 294.12 | 0.002084040 | 89 | 247.39 | 0.020876300 |
| 40 | 286.12 | 0.000415861 | 90 | 247.28 | 0.019408600 |
| 41 | 286.12 | 0.000399333 | 91 | 246.74 | 0.001211440 |
| 42 | 286.01 | 0.000004455 | 92 | 246.53 | 0.002532410 |
| 43 | 276.18 | 0.000947926 | 93 | 246.31 | 0.000281736 |
| 44 | 274.87 | 0.000068727 | 94 | 246.20 | 0.095704600 |
| 45 | 273.89 | 0.027293700 | 95 | 245.45 | 0.021106800 |
| 46 | 273.78 | 0.000024077 | 96 | 245.45 | 0.029352600 |
| 47 | 273.78 | 0.030608000 | 97 | 245.01 | 0.030644700 |
| 48 | 273.67 | 0.000219230 | 98 | 245.01 | 0.021740000 |
| 49 | 272.14 | 0.000999980 | 99 | 243.07 | 0.000001329 |
| 50 | 271.82 | 0.010108700 | 100 | 242.31 | 0.010507800 |


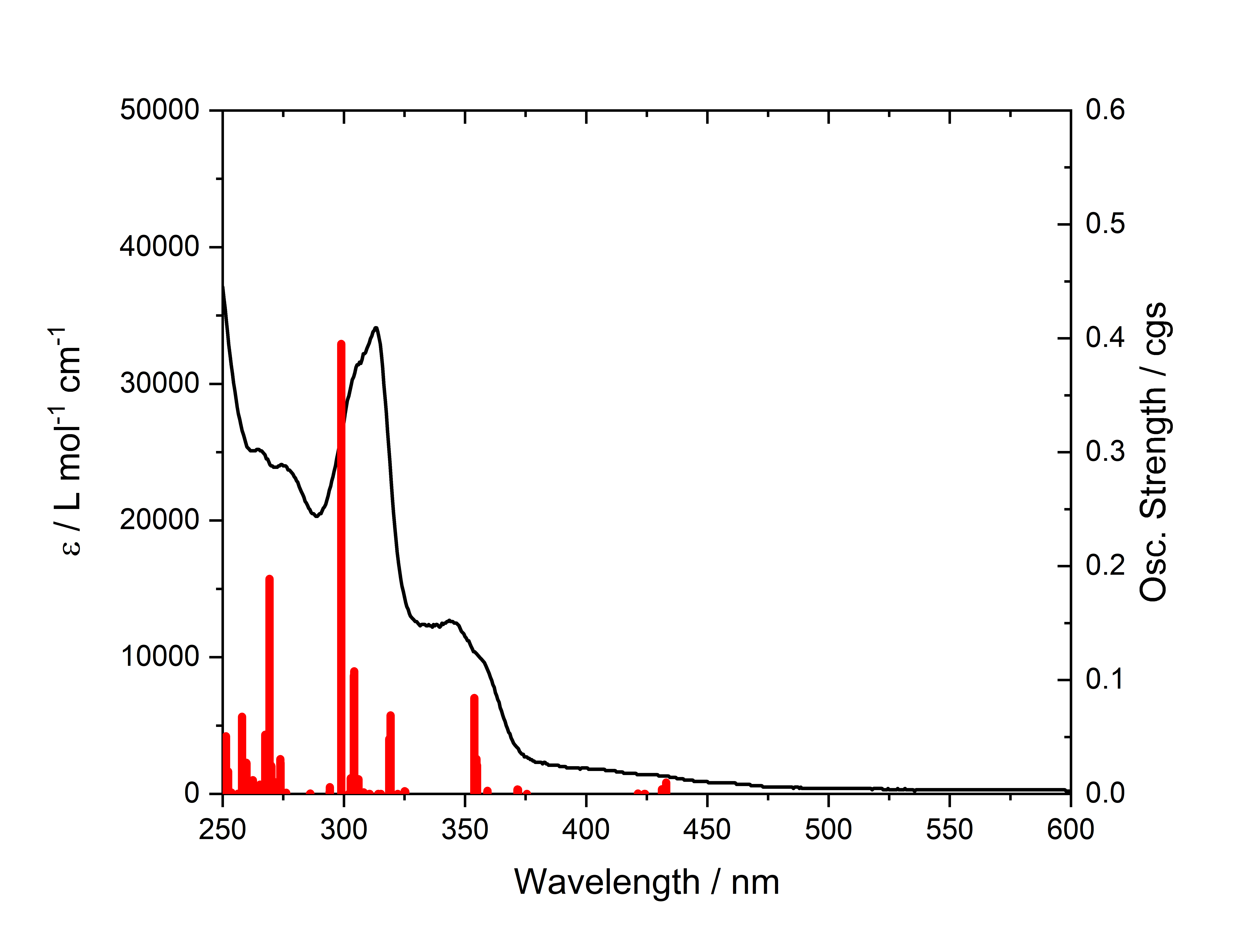


**Figure S6.** Experimental UV-Vis spectrum of compound [Cr(bipy)_3_]^3+^ in acetonitrile and calculated oscillator strength of the calculated electronic transitions.

| 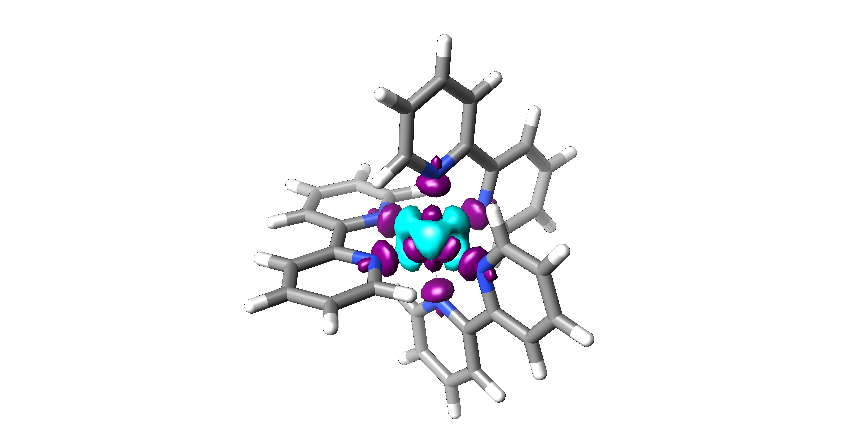  #4 – 424.27 nm - MC | 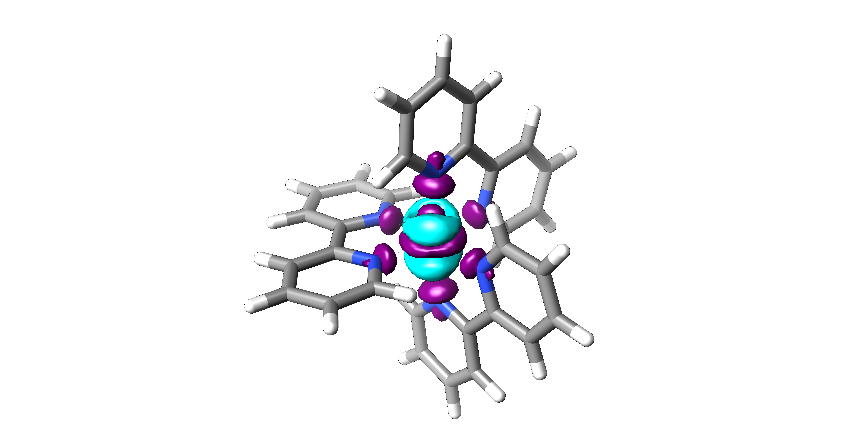  #5 -423.93 nm - MC | 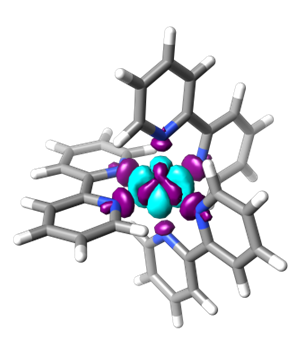  #6 – 421.31 nm - MC |
| --- | --- | --- |
| 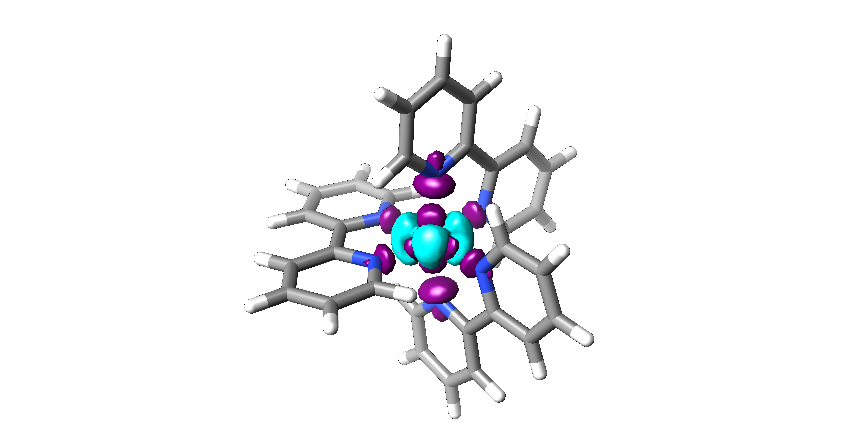  #10 – 359.28 nm - MC | 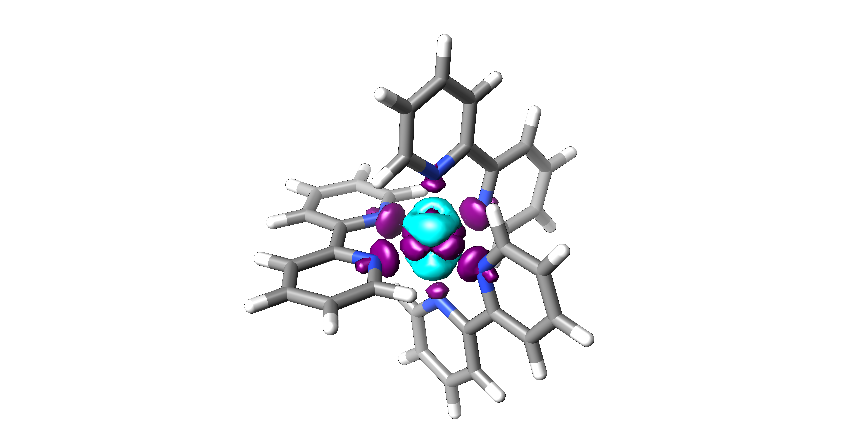  #11 – 359.17 - MC | 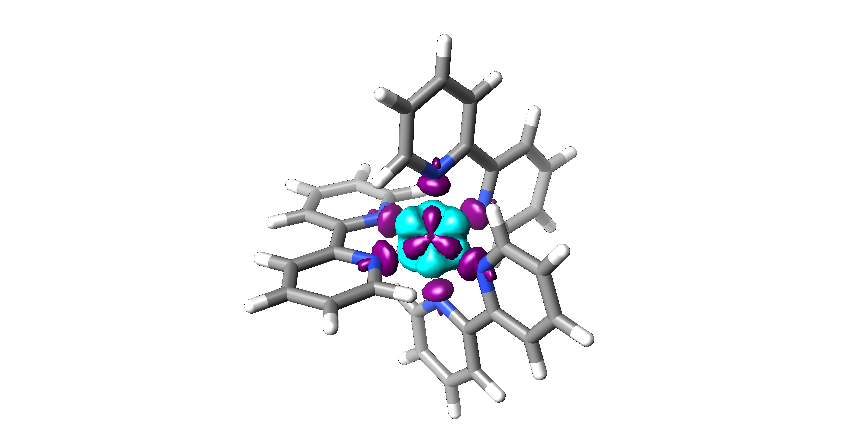  #14 – 354.47 nm -MC |

**Figure S7.** Electron density difference maps (EDDMs) for the metal-centered transitions of [Cr(bipy)_3_]^3+^. Blue: density loss; Purple: density gain.

*Geometries*

[Cr(bipy)_3_]^3+^

Cr 9.00414400849839 15.60231046871831 20.01700098072181

N 9.12710593749272 13.84931141461434 18.94828258091459

C 10.02828697662772 12.95485938506446 19.42222555135082

C 10.18478852831517 11.71678787068482 18.81658174253262

C 9.40736913411207 11.39708789014195 17.71284872625980

C 8.48925772271071 12.32138257533294 17.23665966185216

C 8.37643725513752 13.53816268337807 17.88362447007733

H 10.90004001396782 11.00641049855904 19.19895705772286

H 9.51920907318868 10.43525740411025 17.23306431891969

H 7.86490822677719 12.11275234279527 16.38117615864130

H 7.67633685567804 14.28515784991658 17.54547543322629

N 10.46294862826988 16.58693922163186 18.95445700196181

N 7.42327108236132 16.37527629539245 18.95379348900091

N 7.42326533598655 14.82935034226013 21.08020285443301

N 9.12710390495545 17.35530903246991 21.08572259018013

N 10.46294645259714 14.61768244226517 21.07954947508398

C 10.79682098410417 13.39691900267336 20.59480505176954

C 10.79681403838395 17.80770689372250 19.43919620088228

C 11.10669514425974 16.09483519581218 17.88800781006563

C 6.19841642656885 16.03911373326797 19.42593775301922

C 7.52933597065503 17.17640840988406 17.88564979969399

C 6.19841323266018 15.16551884127835 20.60805620618627

C 7.52932470344290 14.02821621090883 22.14834545105954

C 10.02828484810200 18.24976180405417 20.61178027942587

C 8.37644004455065 17.66645451051743 22.15038499417928

C 11.10669368549838 15.10978927759694 22.14599695845583

C 11.80142601152439 12.64772891939424 21.18982629625887

C 11.80140648067892 18.55690597698615 18.84416512330269

C 12.11377514265868 16.79635743972654 17.25098062519663

H 10.80060839326499 15.12201224145551 17.53795093924520

C 5.04740219988565 16.50572583369399 18.80819243008852

C 6.41849667816080 17.67426986522462 17.22969468755640

H 8.52607800352444 17.41452247422374 17.55076723638285

C 5.04739594360425 14.69891114941134 21.22579849190117

C 6.41848188370201 13.53035786323240 22.80429687943337

H 8.52606508934362 13.79009675788786 22.48322945730337

C 10.18479198527110 19.48782984292792 21.21742955349945

C 8.48926511782624 18.88323168551774 22.79735437862729

H 7.67633989020810 16.91945933441009 22.48853447372760

C 12.11378612154567 14.40827561483411 22.78301454410629

H 10.80059928486209 16.08260784430551 22.49605911104336

C 12.46630457238446 13.15868487428926 22.29500547831021

H 12.06795694291586 11.67932955917556 20.79757016031398

C 12.46628335472282 18.04595382459376 17.73898299712137

H 12.06792853648955 19.52530994111470 19.23641591521181

H 12.60523296621979 16.36294681052047 16.39316913379821

C 5.15901860085000 17.33076610319258 17.69849630792676

H 4.07461712492432 16.23135390521999 19.18367778982308

H 6.54903644136146 18.31515583119049 16.37093144739914

C 5.15900625189141 13.87386791180561 22.33549299871502

H 4.07461303613418 14.97328806049659 20.85031115621400

H 6.54901702588324 12.88946978433311 23.66305924259710

C 9.40737809350052 19.80752621550321 22.32116723008350

H 10.90004415052080 20.19820714039600 20.83505522307354

H 7.86491866252191 19.09186000022545 23.65284058084380

H 12.60524431678755 14.84168846169060 23.64082462243595

H 13.25120280638944 12.58464635948924 22.76644664368149

H 13.25117124285272 18.62000011807365 17.26753429070040

H 4.26952124944833 17.70038479293394 17.20868362797075

H 4.26950607882187 13.50425185829953 22.82530235562087

H 9.51922210441615 20.76935400717243 22.80095597286848

The dipolar magnetic interaction between two magnetic moments can be calculated by the following equation for the energy of the dipole-dipole interaction:^S7^

$$H_{dip}= \frac{{-\mu}_{o}}{4\pi}\frac{1}{r^{3}}\left[ 3\left( \vec{\mu_{i}}\cdot\vec{u_{ij}} \right)\vec{u_{ij}}-\vec{\mu_{i}} \right]\cdot\vec{\mu_{j}}$$

where u_ij_ is the unit vector connecting the interacting centres i and j and *r* is their distance, μ_i,_ and μ_j,_ are the magnetic moments of centers *i* and *j* and μ_0_ is the vacuum permittivity.

Considering only the ground state with parallel anisotropy axes, the energy of the dipole-dipole interaction can be written as follows:

$$H_{dip}= \frac{{-\mu}_{o}}{4\pi}\frac{\mu_{i}\mu_{j}}{r^{3}} \left( 3{cos}^{2}\theta-1 \right)S_{z1}S_{z2}$$

Then, the contribution to of the dipolar interaction to the magnetic coupling constant should be:

$J_{dip}= \frac{\mu_{o}}{4\pi}\frac{\mu_{i}\mu_{j}}{r^{3}} \left( 3{cos}^{2}\theta-1 \right)$

The maximum dipolar antiferromagnetic contribution to the antiferromagnetic coupling was estimated to be *J*_dip_ = -0.002 cm^-1^, using the above equation (when the magnetic moments are collinear and q = 90º).

***References***

[S1] G.M. Sheldrick, SHELXT - Integrated space-group and crystal-structure determination, Acta Crystallogr A 71 (2015) 3–8.

[S2] O. V. Dolomanov, L.J. Bourhis, R.J. Gildea, J.A.K. Howard, H. Puschmann, OLEX2: A complete structure solution, refinement and analysis program, J Appl Crystallogr 42 (2009) 339–341.

[S3] G. M. Sheldrick. Crystal structure refinement with SHELXL. Acta Cryst. (2015). C71, 3-8

[S4] [Lauren L. K. Taylor](https://pubs.rsc.org/en/results?searchtext=Author%3ALauren%20L.%20K.%20Taylor), [Rebecca Andrews](https://pubs.rsc.org/en/results?searchtext=Author%3ARebecca%20Andrews), [April C. Y. Sung](https://pubs.rsc.org/en/results?searchtext=Author%3AApril%20C.%20Y.%20Sung),  [Iñigo J. Vitorica-Yrezabal](https://pubs.rsc.org/en/results?searchtext=Author%3AI%C3%B1igo%20J.%20Vitorica-Yrezabal)a and [Imogen A. Riddell](https://pubs.rsc.org/en/results?searchtext=Author%3AImogen%20A.%20Riddell). Chem. Commun., (2022), 58, 12301-12304

[S5] Huangtianzhi Zhu, Tanya K. Ronson, Kai Wu, and Jonathan R. Nitschke. *J. Am. Chem. Soc.* (2024), 146, 2370−2378

[S6] a) F. Neese, *WIREs Comput. Mol. Sci.* (2012), *2*, 73-78; b) F. Neese, *WIREs Comput. Mol. Sci.* (2022), e1606.

[S7] P. Panissod and M. Drillon, Magnetism: Molecules to Materials IV, (Eds: J. S. Miller and M. Drillon), Wiley-VCH Verlag GmbH & Co. KGaA, Weinheim, 2002, ch. 7, pp. 235.
